# In vivo 5-ethynyluridine (EU) labelling detects reduced transcription in Purkinje cell degeneration mouse mutants, but can itself induce neurodegeneration

**DOI:** 10.1186/s40478-021-01200-y

**Published:** 2021-05-21

**Authors:** Lisanne J. van’t Sant, Joshua J. White, Jan H. J. Hoeijmakers, Wilbert P. Vermeij, Dick Jaarsma

**Affiliations:** 1 Department of Neuroscience, Erasmus University Medical Center Rotterdam, PO Box 2040, 3000 CA Rotterdam, The Netherlands; 2 Department of Molecular Genetics, Erasmus MC Cancer Institute, Erasmus University Medical Center Rotterdam, Rotterdam, The Netherlands; 3 Princess Máxima Center for Pediatric Oncology, Utrecht, The Netherlands; 4 Oncode Institute, Utrecht, The Netherlands; 5 Institute for Genome Stability in Ageing and Disease, CECAD Research Centre, Cologne, Germany

**Keywords:** Purkinje cell degeneration, 5-Ethynylurdine, Nascent RNA, Transcription stress, DNA damage, Ercc1, SCA1

## Abstract

Fluorescent staining of newly transcribed RNA via metabolic labelling with 5-ethynyluridine (EU) and click chemistry enables visualisation of changes in transcription, such as in conditions of cellular stress. Here, we tested whether EU labelling can be used to examine transcription in vivo in mouse models of nervous system disorders. We show that injection of EU directly into the cerebellum results in reproducible labelling of newly transcribed RNA in cerebellar neurons and glia, with cell type-specific differences in relative labelling intensities, such as Purkinje cells exhibiting the highest levels. We also observed EU-labelling accumulating into cytoplasmic inclusions, indicating that EU, like other modified uridines, may introduce non-physiological properties in labelled RNAs. Additionally, we found that EU induces Purkinje cell degeneration nine days after EU injection, suggesting that EU incorporation not only results in abnormal RNA transcripts, but also eventually becomes neurotoxic in highly transcriptionally-active neurons. However, short post-injection intervals of EU labelling in both a Purkinje cell-specific DNA repair-deficient mouse model and a mouse model of spinocerebellar ataxia 1 revealed reduced transcription in Purkinje cells compared to controls. We combined EU labelling with immunohistology to correlate altered EU staining with pathological markers, such as genotoxic signalling factors. These data indicate that the EU-labelling method provided here can be used to identify changes in transcription in vivo in nervous system disease models.

**Supplementary Information:**

The online version contains supplementary material available at 10.1186/s40478-021-01200-y.

## Introduction

5-Ethynyluridine (EU) and other modified cell-permeable nucleosides, such as 4-thiouridine and bromo-uridine, have been used to label newly synthesized RNA [29, 38, 77]. The uridine analogues are taken up by cells, converted to nucleotide phosphates, and then incorporated into nascent RNAs by all three RNA polymerases [29]. These metabolic RNA labelling methods have been used to study the dynamics of RNA production and degradation [14, 54, 62, 68], to characterize newly generated RNA transcripts, and to capture nascent RNA binding proteins [3, 20]. EU labelling of nascent RNA, which is detected via attachment of fluorophores or biotin by using click chemistry [3, 29, 33], also enables microscopic analysis of the distribution of labelled RNA [29, 45, 55, 62]. Microscopic analysis of EU labelling has been used to monitor dynamics of transcription [62], global changes in RNA synthesis following DNA damage [4, 8, 39, 44, 79] or changes in RNA expression following drug treatment [17–19, 31, 53]. EU labelling of nascent RNA has also been employed in human organotypic slice cultures [41, 52], snail nervous system preparation [23], and living animals, including juvenile snail [23], zebrafish larvae [1], and mice [29, 39, 41], all studies indicating substantial heterogeneity in EU labelling between cell types, tissues, and age. In addition, EU labelling has been applied to neurons, revealing that in cultured mouse hippocampal neurons and zebrafish larval nervous system EU labelled RNA may accumulate in dendrites, and that altered neuronal activity influences the amount of EU labelling [1].

To further test EU labelling in neurons and to evaluate whether the method can be used in mouse models of nervous system disorders, in the present study, we applied EU labelling in the mouse central nervous system in vivo. In previous in vivo mouse studies, EU has been injected intraperitoneally [29, 39], but this method of application resulted in low levels of EU labelling in the nervous system. We therefore injected EU directly into the brain parenchyma, focusing on the cerebellar cortex because of its relatively simple, well-characterized histoarchitecture. Injections of EU in the cerebellum resulted in reproducible nuclear labelling of all cerebellar cell types. The different cell types showed distinct relative intensities and temporal changes in EU labelling, with Purkinje cells showing the highest level of labelling that persisted for more than two weeks. We also found that Purkinje cells developed pathological structures and started to degenerate and die beginning 9 days post-injection. Finally, we examined EU labelling in the cerebellum of two Purkinje cell-specific mutant mouse models for, respectively, a severe progeroid DNA repair deficiency syndrome (*Ercc1*^*d*/*f*^*Pcp2-Cre* mice) [11] and spinocerebellar ataxia 1 (SCA1, ATXN1[82Q] mice) [7]. In both mouse models, we found reduced EU-labelling intensities consistent with transcriptional abnormalities. Our data show that although EU may be neurotoxic for Purkinje cells in the long-term, the EU labelling method can be used to investigate changes in transcription in the central nervous system in vivo using short post-injection survival times.

## Method

### Animals

Animal experiments were performed according to institutional guidelines as overseen by the Animal Welfare Board of the Erasmus MC, following Dutch and EU legislation. Prior to the start of the experiments, a project license for the animal experiments performed for this study was obtained from the Dutch national authority and filed under no. AVD101002015273. Mice were group-housed in standard cages at 20–22 °C and 12:12 light/dark cycle with ad libitum access to water and standard laboratory food. Male and female C57Bl6/J mice, aged 2–6 months, were used as wild-type mice.

*Ercc1*^*d/f*^*Pcp2-Cre* mice express a truncated “delta” *Ercc1* allele (*Ercc1*^*d*^; *Ercc1*^*tm2Jhjh*^ in MGI database) [12, 76], a “floxed” Ercc1 allele surrounded by loxP sites (E*rcc1*^*f*^; *Ercc1*^*tm2Dwm*^) [5, 13], and a *Pcp2-Cre* transgene that drives Cre recombinase expression specifically in postnatal Purkinje cells [11]. After recombination by Cre, the floxed *Ercc1* allele becomes a null allele, resulting in Purkinje cells that have one null and one truncated *Ercc1* allele, leading to a less than 10% of normal ERCC1 levels [76]. Female *Ercc1*^*d/*+^*Pcp2-Cre* mice in C57Bl6/J background were generated, via crossing of female *Pcp2-Cre* mice with male *Ercc1*^*d/*+^ mice, and subsequently these female *Pcp2-Cre*^+^*/Ercc1*^*d/*+^ were crossed with male *Ercc1*^*f/f*^ mice in FVB background to yield *Ercc1*^*d/f*^*Pcp2-Cre*^+^ mice in a uniform C57BL6J/FVB F1 hybrid background. *Ercc1*^+*/f*^*Pcp2-Cre*^+^ and *Ercc1*^*d/f*^*Pcp2-Cre*^*−*^ transgenic mice that do not develop a degenerative phenotype served as controls.

ATXN1[82Q] transgenic mice were kindly provided by Dr. Harry T. Orr (University of Minnesota, Minneapolis, MN, USA) and were derived from the B05 line that overexpresses human ATXN1 cDNA containing an 82 CAG-repeat under the Purkinje cell-specific Pcp2 promoter (Tg(Pcp2-ATXN1*82Q)5Horr) [7]. The mice were F1 offspring from crossings between ATXN1[82Q] mice on the FVB/NHsd background and C57Bl6/J mice. Non-transgenic littermates were used as controls.

### Intracerebellar 5-ethynyluridine (EU) injections

To inject EU (Jena Bioscience, CLK-N002-10) in the cerebellar cortex, we followed standard surgical procedures for stereotaxic injections [25]. Mice were anesthetized with a mixture of isoflurane/oxygen (5% for induction, 1.5–2.0% for maintenance), while carprofen (Rimadyl Cattle i.p. 5 mg/kg), buprenorphine (Temgesic, i.p. 0.05 mg/kg), lidocaine (s.c. 0.4 mg/ml) and bupivacaïne (s.c. 0.1 mg/ml) were injected to reduce peri-surgical pain and inflammation. Ophthalmic ointment was applied to the eyes to prevent corneal drying and damage. Body temperature was monitored and kept constant at 37 °C throughout the surgical procedure. Mice were positioned in a stereotaxic frame (Stoelting Co., Wood Dale IL, USA), and the cerebellar surface was exposed by removing overlying skin and neck muscles and drilling a craniotomy (diameter ~ 1 mm) in the occipital bone. Injections were made using a glass micropipette (tip diameter 10 µm) centred in the caudal vermis based on coordinates from the Paxinos mouse brain atlas [51]: anterior–posterior − 6.48 mm, lateral 0 mm, dorsoventral − 1.4 mm from bregma. 1 μl of EU (75 mM in PBS) was injected using mechanical pressure at a rate of 10 nl/s. The micropipette was held in place for 10 min following the end of injection before retraction to allow sufficient diffusion of injected solution, and the skin overlying the occipital bone was sutured. To examine the evolution in the distribution of EU labelling in time, animals were sacrificed at various time points following retraction of the micropipette, ranging from 10 min to 2 weeks. In experiments with Purkinje cell degeneration mouse models and their control littermates, animals were sacrificed 1 h after the injection. To control for the specificity of EU labelling and unanticipated effects of the injection procedure, a subset of mice were injected with 1 μl PBS instead of EU. To show that EU labelling indeed represents newly produced RNA, another subset of mice received an intracerebellar micro-injection of the transcription inhibitor actinomycin D (0,5 μl of 6.25 μg/μl solved in saline, Sigma-Aldrich) 1 h prior to EU injection with a post-EU-injection survival time of 1 h.

### EU staining and histological procedures

To visualize EU, mice were deeply anaesthetized with an overdose of pentobarbital and perfused transcardially with 20 ml 0.9% saline followed by 50 ml 4% paraformaldehyde (PFA) in 0.12 M phosphate buffer, pH 7.4. Brains were carefully removed and post-fixed for 1 h in 4% PFA at room temperature, then placed in 10% sucrose overnight at 4 °C. Multiple brains were embedded together in 12% gelatin blocks [5], and incubated overnight in 30% sucrose. Brains were rapidly frozen and sectioned coronally at 40 μm using a freezing microtome (SM2000R, Leica). Sections were serially collected in 8 vials, such that each vial contained a series of sections throughout the anterior–posterior axis of the cerebellum with intervals of 320 μm.

Routinely, one series was processed for EU detection with click chemistry using Alexa A555-azide (ThermoFisher, A20012) and a commercial kit (Click-iT, ThermoFisher, C10276). Sections were stained for 30 min, washed and counterstained with DAPI (4’­6-diamidino-2-phenylindole, 1 μg/ml in PBS). Other series were processed with a combination of click chemistry for EU detection and immunofluorescence for cell type and neuropathological markers. Sections were rinsed with phosphate buffered saline (PBS) pH7.4, preincubated in PBS containing 0.4% Triton X‐100 (PBST) and 10% normal horse serum (NHS) for 1 h at room temperature, and incubated for 48 h at 4 °C with different combinations of primary antibodies (see Table 1) in PBST with 2% NHS. Sections were subsequently processed for the click reaction with Alexa A555-azide, and then incubated with secondary antibodies raised in donkey, diluted 1:400 in PBST 2% NHS, carrying Alexa Fluor A488 or A647 as fluorophores (Jackson Immunoresearch). Finally, sections were stained with DAPI to visualize cell nuclei, and mounted on coverslips, and placed on glass slides with Mowiol mounting medium.

**Table 1:** Antibodies

Antibodies (dilution)	Source	Identifier (Cat. No; RRID)
Rabbit anti-ATF3 (1:2000)	SantaCruz	SC-188; AB_2258513
Rabbit anti-Calbindin (1:10,000)	Swant	CB-38a; AB_10000340
Rabbit anti-Cleaved caspase 3 (Asp175) (1:500)	Cell signaling technology	9661; AB_2341188
Rabbit anti-GABARAPL1 (1:1000)	Proteintech	11010-1-AP; AB_2294415
Rabbit anti-Iba1 (1:1000)	Wako	019-19741; AB_839504
Rabbit anti-IP3 receptor (1:4000)	Abcam	ab5804; AB_305124
Rabbit anti-LaminB1 (1:500)	Abcam	ab16048; AB_443298
Rat anti- LGALS3 (Mac2; 1:2000)	Cedarlane	8942AP; AB_10060357
Rabbit anti-MacroH2A1 (1:250)	Upstate biotechnology	#07–219; AB_11214187
Mouse anti-NeuN (1:2000)	Millipore	MAB377; AB_2298772
Mouse anti-Nucleoporinp62 (1:1000)	BD Pharmingen	BD 610497; AB_397863
Rabbit anti-P53 (1:2000)	Leica	P53-CM5P; AB_2744683
Mouse anti-Parvalbumin (1:1000)	Swant	235; AB_10000343
Rabbit anti-S100 (1:500)	Abcam	76729; AB_1524357
Mouse anti-p62/SQSTM1 (1:2000)	Abcam	56416; AB_945626
Mouse anti-SF3a 120 (1:500)	Synaptic systems	204 011; AB_887898
Rabbit anti-Sumo1 (1:500)	Sigma-Aldrich	SAB4503055; AB_10746310
Rabbit anti-Ubiquitin (1:2000)	Dako	Z0458; AB_2315524
Mouse anti-Ubiquitin/fk2 (1:1000)	Enzo Life Sciences	BML-PW8810-0500; AB_2051891
Mouse anti-γH2AX (1:500)	Millipore	05–636; AB_309864

### Image acquisition and analyses

**Fig. 1:**
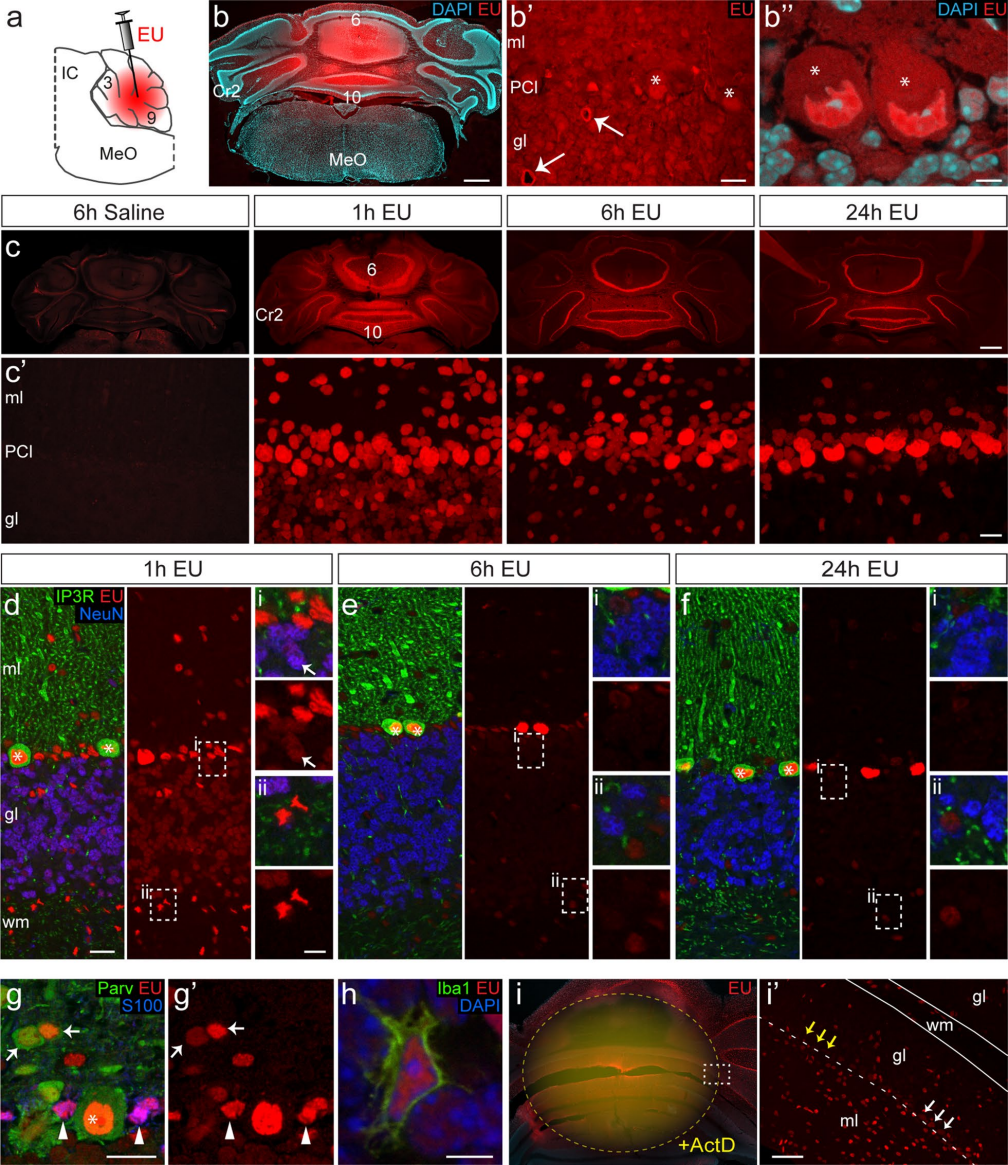
Differential labelling of cerebellar cell types following EU injection. **a** Schematic representation of intracerebellar EU injection (midsagittal caudal mouse brain). **b** Widefield tile scan (**b**) and confocal images (**b′**, **b″**, 1-μm optical section) of coronal cerebellar sections of EU-injected mouse paraformaldehyde-fixed 10 min after termination of the injection. EU, visualized by click chemistry with Alexa555-azide, is widely distributed throughout the caudal vermis, with decreasing levels of EU in the lateral cerebellar hemispheres. EU-labelling fills the entire neuropil with slightly higher levels in the nuclei of Purkinje cells (asterisks in **b′** and **b″**) and the walls of blood vessels (arrow in **b′**). **c** Widefield tile scans (**c**) and confocal images (**c′**, maximal projection of 20 images, 9-μm stacks) of cerebellar cortex of EU or saline injected animals perfused 1 h, 6 h or 24 h following EU injection. Note the absence of labelling in saline injected animals, and abundant nuclear EU labelling in all layers in EU injected animals. Further note, subtle differences in EU labelling at different post-injection time points, in particular in the granule cell layer (gl), where all cells are labelled at 1 h, while only a fraction of cells is labelled at 6 h and 24 h. **d**–**h** Confocal images (1-μm optical sections) of combined EU staining and immunofluorescence of cellular markers: Inositol trisphosphate receptor (IP3R), Purkinje cells; Neuronal nuclei antigen (NeuN), granule cells (arrow in **d**); Parvalbumin (Parv), molecular layer (ml) interneurons (arrows in **g**) and Purkinje cells; S100β, Bergmann glia (arrow heads in **g**) and other astrocytes; Iba1, microglia (**h**). **i** Widefield tile scan (**i**) and confocal image (**i′**, thickness optical section 9 μm) of EU labelling 1 h after EU-injection in a mouse that received an actinomycin D (ActD) injection 1 h prior to the EU injection in the same site. The yellow area (**i**) marks the area with either no or strongly reduced EU staining surrounding the ActD injection site. The arrows point to unlabelled (yellow) and weakly labelled (white) Purkinje nuclei. Scale bars: 500 μm (**b**, **c**), 50 μm (**i′**), 20 μm (**b′**, **c**, **d**, **g**) and 5 μm (**b″**, **h**, insert in **d**). 3, 6, 9 and 10, lobules 3, 6, 9 and 10 of cerebellar cortex; Cr2, Crus2 of cerebellar cortex; IC, inferior colliculus; MeO, medulla oblongata; PCl, Purkinje cell layer; wm, white matter

Overview images of EU fluorescent labelling were collected using a ZEISS Axio Imager M2 fluorescence microscope with Plan-Apochromat 10 × objective and ZEN software for image acquisition and stitching (see Fig. 1 for example images). We imaged at least one series of coronal sections per mouse (this is every 8th section; interval between sections 320 μm) to map the distribution and intensity of EU labelling throughout the cerebellum. Sections from all mice were imaged using the same microscope and camera settings. For each of these sections, we measured the area with labelled cells. By multiplying the sum of areas from a series by 320 μm, we obtained an estimate of the volume of cerebellum containing EU labelled cells.

Cellular and subcellular distributions of EU fluorescent signal were analysed and imaged with a Zeiss LSM700 confocal laser scanning microscope with a Plan-Apochromat 63x/1.40 Oil objective. We collected stacks of 10–20 optical sections, 1 μm-thick, with 0.1 μm x–y resolution and z-steps of 0.3 or 0.45 μm, yielding stacks of 102 × 102 × 3 to 9 (xyz) μm. For images with larger x–y dimensions, we used the tilescan function. For most analyses, laser intensity and gain were set to avoid pixel saturation of EU signal in the nuclei of Purkinje cells, representing the most intensely labelled structures. However, for the analysis of cytoplasmic staining in Purkinje cells, or lightly labelled cells, such as cerebellar granule cells, we also acquired images with saturated signal in Purkinje cell nuclei. To enable direct comparison of EU labelling of cells in different layers, we collected stacks in a plane perpendicular to the Purkinje cell layer, to have all layers and representative examples of cell types in the same stack. In view of their asymmetric morphology, having a flattened (and puckered) side facing the dendritic tree [50], for the analysis of EU labelling in the nuclei of Purkinje cells we also acquired stacks in a plane parallel to the Purkinje cell layer. This orientation also results in a higher number of complete Purkinje cell nuclei in the same stack (e.g. see Fig. 7a, b), which, for instance, facilitated the measurements of nuclear volumes in ATXN1[82Q] mice.

**Fig. 2:**
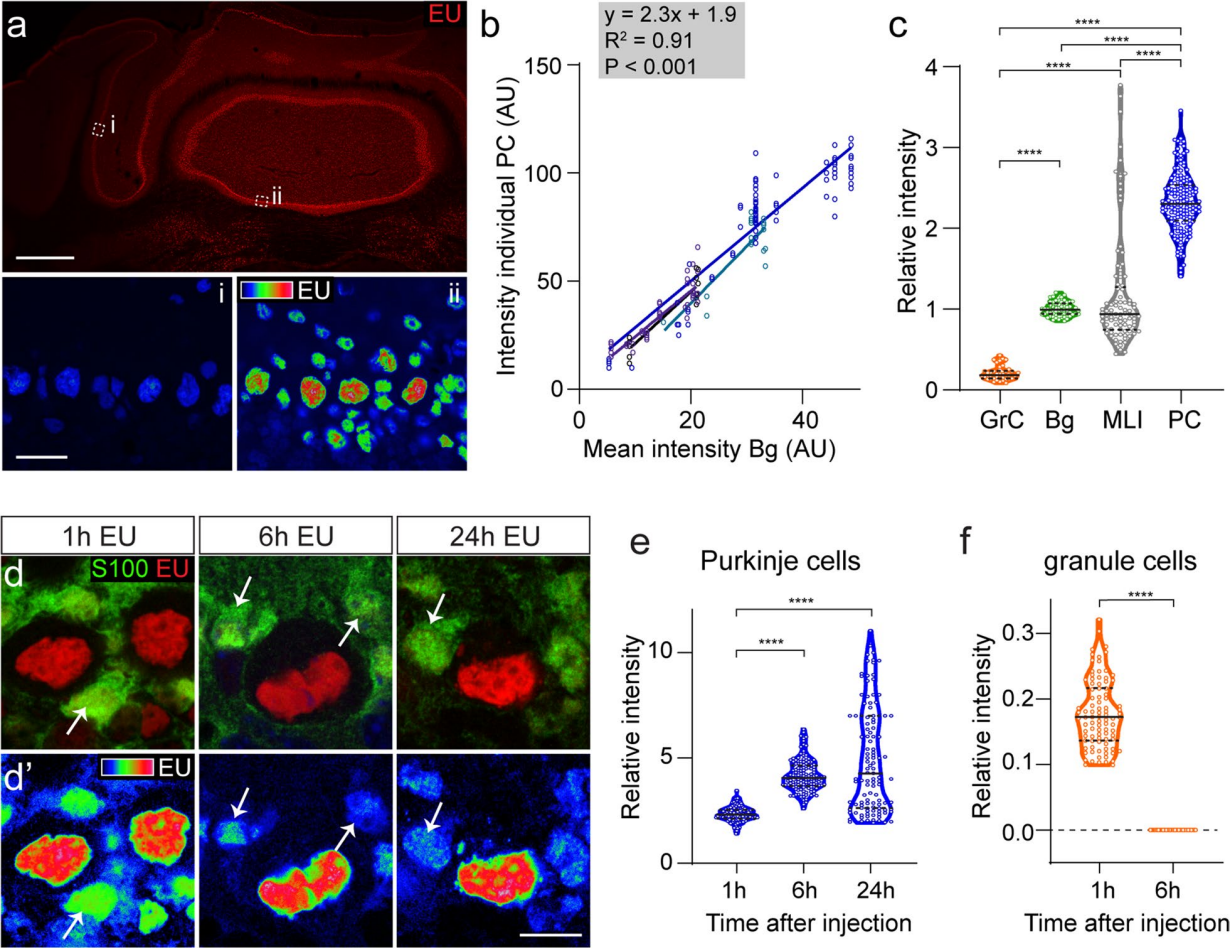
Analysis of relative EU-intensities in different cerebellar cell types. **a** EU labelling intensity depends on the distance from the EU injection site. Upper image is a widefield tile scan. Lower images are color-coded high magnification confocal images of highlighted areas, illustrating lower EU intensities in the Purkinje cell layer in lateral (**a′**) versus medial (**a″**) cerebellar cortex. **b** EU labelling intensities of individual Purkinje cells plotted against the mean intensities of surrounding Bergman glia. Values show a linear relationship between these values over a substantial interval of labelling intensities. Data points and linear regression lines are from four animals (3 coronal sections per animal, > 3 regions per section). Linear regression parameters in the grey box are based on all values. **c** Violin plot showing relative EU labelling intensities (= labelling intensity divided by mean labelling intensity of nearby Bergman glia cells) of different cerebellar cell types. **d**, **e** Confocal images (maximal projection of 6 images, 3-μm stacks), color-coded representation of EU-signal (**d′**), and violin plots illustrating increased relative EU labelling intensity in Purkinje cells 6 and 24 h post-injection. Nuclei of Bergman glia cells are marked by S100β (arrows in **d** and **d′**). Each dot in **e** represents the labelling intensity in a Purkinje cell divided by the mean of labelling intensity of surrounding Bergman glia cells. **f** Violin plots illustrating EU signal in granule cells relative to Bergman glia cell 1 and 6 h post injection, illustrating that signal in granule cells is absent after 6 h. Horizontal lines indicate the median and the interquartile range (25–75% data range). ****c, **e** *P* < 0.0001. (Kruskal–Wallis test with Dunn’s multiple comparison tests); ****f *P* < 0.0001 (Welch’s t test). Scale bars: 500 μm (**a**); 20 μm (**a′**); 10 μm (**d′**)

Quantitative image analyses were performed using ImageJ software from Fiji [59, 60]. The 3D Object Counter tool was used to identify and measure the mean grey values of the nuclei of Purkinje cells and Bergmann glia cells. EU signal in nuclei of molecular layer interneurons and granule cells were analysed in sections co-stained for the cellular markers parvalbumin or NeuN, respectively, to serve as a guide for drawing the contours of their nuclei. Final mean grey values of cells were obtained after subtraction of background grey value determined in areas without labelling in the same area. Finally, mean grey values of cells were converted into relative grey values by dividing the value by the mean grey values of Bergmann glia cells in the same image. We performed this normalization for several reasons. First, due to the regional heterogeneity of overall labelling intensities (resulting from the focal EU injection), absolute grey values are too variable to be meaningful. Second, Bergmann glia cell are present in all images, and show a relatively constant EU signal with intensities between Purkinje cells and other cell types. Third, grey values in Bergmann glia showed a linear correlation with grey values of adjacent Purkinje cells over a wide range of overall labelling levels (see Fig. 2b). For all analyses, we collected data from at least 3 mice, with at least three sections and multiple sampling sites per mouse, unless otherwise indicated.

Qualitative analysis of EU-labelling in P53 +, γH2AX + and ATF3 + Purkinje cells in *Ercc1*^*d/f*^*Pcp2-Cre* mice was performed by two independent observers (L.v.S. and D.J.) using the same series of confocal images and yielding the same results. 40 P53 + and ATF3 + cells, and 10 γH2AX + were sampled from sections from 4 *Ercc1*^*d/f*^*Pcp2-Cre* mice (> 8 and 2 cells per animal, respectively). EU labelling in P53 + , ATF3 + and γH2AX + Purkinje was designated ‘reduced’ when labelling was distinctly lower than in surrounding Purkinje cells.

The 3D Object Counter tool also was used to obtain estimates of nuclear volumes of ATXN1[82Q] and control Purkinje cells. In ATXN1[82Q] Purkinje cells, we also measured the relative EU signal in the nucleolus compared to the rest of the nucleoplasm. The nucleoli were defined on the basis of intense EU signal flanked by one or two heterochromatin bodies [50].

Purkinje cells with p62 + /EU + bodies in their cytoplasm (Fig. 4o) were manually counted in 2 animals per post-injection survival time, focusing on parts of the cerebellar cortex with the most intense EU labelling. Values are expressed as percentage of the total number of counted cells.

Confocal images presented in this manuscript are derived from maximal projections or single optical sections. Adobe Photoshop (CC2018; Adobe Systems, San Jose, CA) was used to adjust image contrast and assemble the figures.

### Statistical analyses

Statistical analyses were performed using GraphPad Prism version 8.3.0 for Windows (GraphPad Software, La Jolla California USA). Values in graphs either are from individual cells pooled from multiple mice, or represent mean values/mouse. Values in graphs are shown as means ± standard error, or in case of violin plots, by the median and quartiles. For statistical analysis we set the value of α = 0.05, and significance levels were expressed as *P < 0.05, **P < 0.01, ***P < 0.001, and ****P < 0.0001. For comparisons between two groups, we performed unpaired two-tailed Student’s *t*-tests, or Welch's unequal variances *t*-tests. In case of multiple groups, we used one-way ANOVA with Dunn's multiple comparisons test. Linear regression analysis was used to evaluate the correlation between EU intensity of individual Purkinje cells around the mean EU intensity of surrounding Bergmann glia cells.

## Results

### Cerebellar EU injections result in differential intensities and temporal changes of EU labelling between cell types

In previous in vivo studies in mice, EU was injected intraperitoneally (330–750 μmol/g animal), resulting in labelling of nascent RNA in cells of multiple organs and tissues, but not in the central nervous system [29, 39]. To obtain labelling of central nervous system cells, we injected EU (75 nmol in 1 μl saline) directly into the cerebellar cortex (Fig. 1a). The cerebellar cortex has a relatively simple laminar histoarchitecture, dominated on the one hand by a layer of Purkinje cells, which are large, metabolically and transcriptionally highly active neurons, and on the other hand by a massive amount of granule cells, that are densely packed in granule cell layer, and are among the smallest neurons in the nervous system [73]. Animals were perfused with fixative 10 min or 1, 6, or 24 h post-EU injection, and cerebellar sections were processed for copper (I)-catalysed cycloaddition reaction (click chemistry) with Alexa A555-azide to visualize EU labelled nascent RNA (Fig. 1b, c). The 10 min timepoint (n = 2) served to examine the distribution of EU shortly after the termination of injection (Fig. 1b), while the other time points (n = 4 animals/time point) represent incubation times used in previous studies [1, 29, 39]. Animals injected with saline only showed no or minimal staining following the click reaction and served as controls (Fig. 1c).

The injections resulted in EU labelling in a significant portion of the cerebellum, with the highest staining intensities closest to the site of injection in the caudal vermis (lobules VI-X), and lower staining intensities or no staining in parts of the cerebellar cortex more distant from the injection site, i.e. lateral and rostral aspects of the cerebellar cortex (Fig. 1b, c). The volume of cerebellum containing detectable EU labelling varied from 26 to 33 mm^3^, and did not significantly differ in animals sacrificed at different post-injection times (One-way ANOVA, F[4, 5]  = 0.58; *P* = 0.68). In particular, the volume with labelling was similar at 10 min (27 ± 0.6 mm^3^) and 1 h (29 ± 0.2 mm^3^), indicating that injected EU has spread throughout the cerebellar parenchyma within 10 min after injection.

After 10 min, EU labelling showed a diffuse distribution throughout the neuropil with somewhat increased staining in the nuclei of Purkinje cells, as well as walls of large vessels (Fig. 1b). In contrast, 1 h post-injection, visible EU labelling was only present in nuclei (Fig. 1c), consistent with incorporation in nascent RNA [29]. Labelled nuclei were apparent in all layers of the cerebellar cortex, including the white matter, again validating EU distribution across the entire cerebellar parenchyma. Morphological analyses and double labelling with specific cellular markers indicated that all cerebellar cell types were labelled (Fig. 1c–g). However, different cell types showed differential staining intensities and temporal changes in EU labelling, with Purkinje cells showing the highest level of labelling at all post-injection time points (Fig. 1c–f). Granule cells showed the lowest staining intensities 1 h post-injection and showed no labelling above background at 6 and 24 h (Fig. 1c–f). Intermediate staining levels were apparent in Bergmann glia, interneurons in the molecular and granular layers, microglia, and oligodendrocytes in the white matter (Fig. 1c–h). No or strongly reduced EU staining occurred in animals injected with the transcription inhibitor Actinomycin D (3.25 μg in 0.5 μl saline) 1 h prior to EU injection (Fig. 1i) consistent with previous studies [29, 39].

In cultured neuronal and non-neuronal cells, the intensity of EU labelling depends on EU concentration: no detectable staining at a concentration of 50 μM, light staining at 200 μM, and maximal staining at 5–10 mM [1, 29]. We anticipated that our focal injections of 75 nmol (in 1 μl), would transiently yield concentrations in the same range, varying from low millimolar levels near the injection site to high micromolar levels more distally. The gradient of labelling intensities observed in our mice is consistent with this notion (Figs. 1, 2a). To examine whether the regional differences in EU labelling intensities impacts on the relative differences between cell types, we systematically examined EU fluorescence intensities of major cell types (Purkinje cells, Bergmann glia cells, granule cells, and molecular layer interneurons) in areas with different overall labelling intensities, and normalized values of individual cells for mean labelling intensities of Bergmann glia in that area. As shown in Fig. 2b, EU labelling intensity of Purkinje cells and Bergman glia showed a linear correlation over a range of labelling intensities (R^2^ = 0.91; *P* < 0.001). The ratio of labelling in granule cells also was relatively constant independent of sampling area or experimental animal, while labelling in molecular interneurons was more variable (Fig. 2c). Relative intensity for EU labelling in cell types depended on post-injection survival time (Fig. 2d–f). Thus, 1 h post-injection, the relative amount of labelling in Purkinje cells was about twofold the labelling levels in surrounding Bergmann glia, and was more than fourfold the amount of labelling in Bergman glia cells 6 and 24 h post injections (Fig. 2d, e). In contrast, granule cells consistently showed one-fifth of the labelling of Bergmann glia cells in animals sacrificed 1 h post injection, and no detectable labelling at later time points (Fig. 2c, f). Together, the data indicate that intracerebellar EU injections result in reproducible nuclear labelling of all cerebellar cell types, with cell type-specific relative intensities and temporal changes.

### Cell type-specific and time-dependent changes in subnuclear distribution of EU labelling

**Fig. 3:**
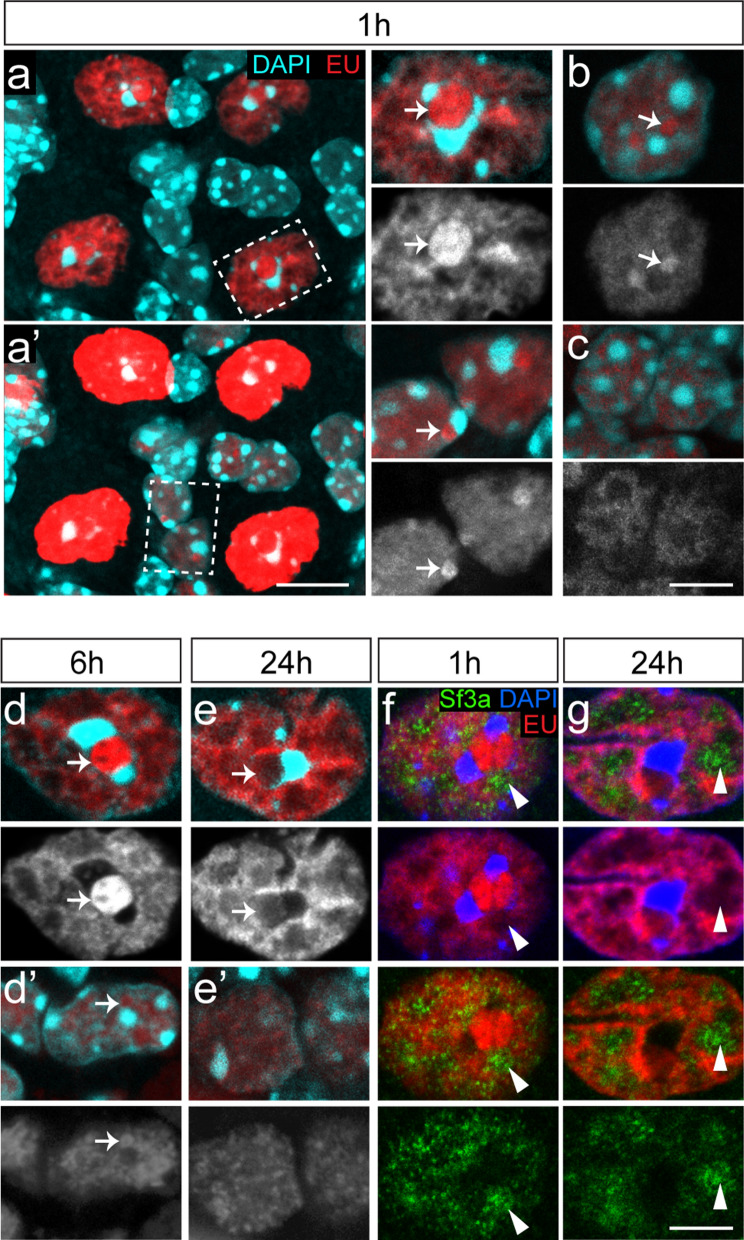
Subcellular distribution of EU in cerebellar neurons. **a**–**e** Representative high magnification confocal images (optical section 1 μm) of EU labelling in nuclei of Purkinje cells (**a**, **d**, **e**), Bergmann glia (**a′**, **d′**, **e′**), a molecular layer interneuron (**b**) and granule cells (**c**) at 1, 6 and 24 h after EU injection. Relative higher levels of EU labelling occur in nucleoli of Purkinje cells and other cerebellar cell types 1 h post injection (arrows in **a**–**d**). No increased nucleolar EU-labelling was found 24 h post-injection (arrow in **e**). Note that image in **a′** is the same as in a but collected with different detector settings resulting in overexposure of Purkinje cells, but better visualisation of EU signal in Bergmann glia. **f**, **g** Co-staining of EU, DAPI and Sf3a showing lower EU signal in DAPI-/Sf3a + domains of the nucleoplasm (arrow heads). Scale bars: 10 μm (**a′**) and 5 μm (**c**, **g**)

Analysis of the subnuclear distribution of EU showed that, 1 h post-injection, labelling was higher in the nucleolus compared to the rest of the nucleoplasm in different cell types, including Purkinje cells (Fig. 3a), Bergman glia cells and molecular interneurons (Fig. 3a, b), but not granule cells (Fig. 3c). This is consistent with previous studies showing dominant EU labelling in nucleoli, representing rRNA transcribed by RNA polymerase I [29]. However, the relative intensity of EU labelling in the nucleoli changed with longer post-injection times. In Purkinje cells, nucleolar EU labelling, while still being higher 6 h post-injection, was lower compared to rest of the nucleoplasm after 24 h (Fig. 3d, e). Similarly, in Bergman glia and molecular layer interneurons EU labelling did not distinctly outline the nucleoli 6 and 24 h post-injection, indicative of a relative reduction as compared to the rest of the nucleoplasm.

In all cells at all time points, no or lower levels of EU labelling was present in nuclear domains that are strongly labelled with DAPI, and are dominated by condensed heterochromatin (Fig. 3), i.e. regions with less active transcription. In addition, Purkinje cell nuclei showed irregular DAPI-negative nuclear domains with lower EU signal (Fig. 3). These domains showed increased immunoreactivity for the splicing factor Sf3a (Fig. 3f, g), indicative of nuclear speckles (also termed interchromatin granule clusters) that are enriched in snRNPs (small nuclear ribonucleoprotein particles) and other splicing factors [65], and that are relatively large in neurons such as Purkinje cells [35]. Reduced labelling of nuclear speckles in Purkinje cells was evident at all three post-injection time points examined (Fig. 3f, g).

### *Cytosolic EU accumulates in p62/SQSTM1* + *bodies*

**Fig. 4:**
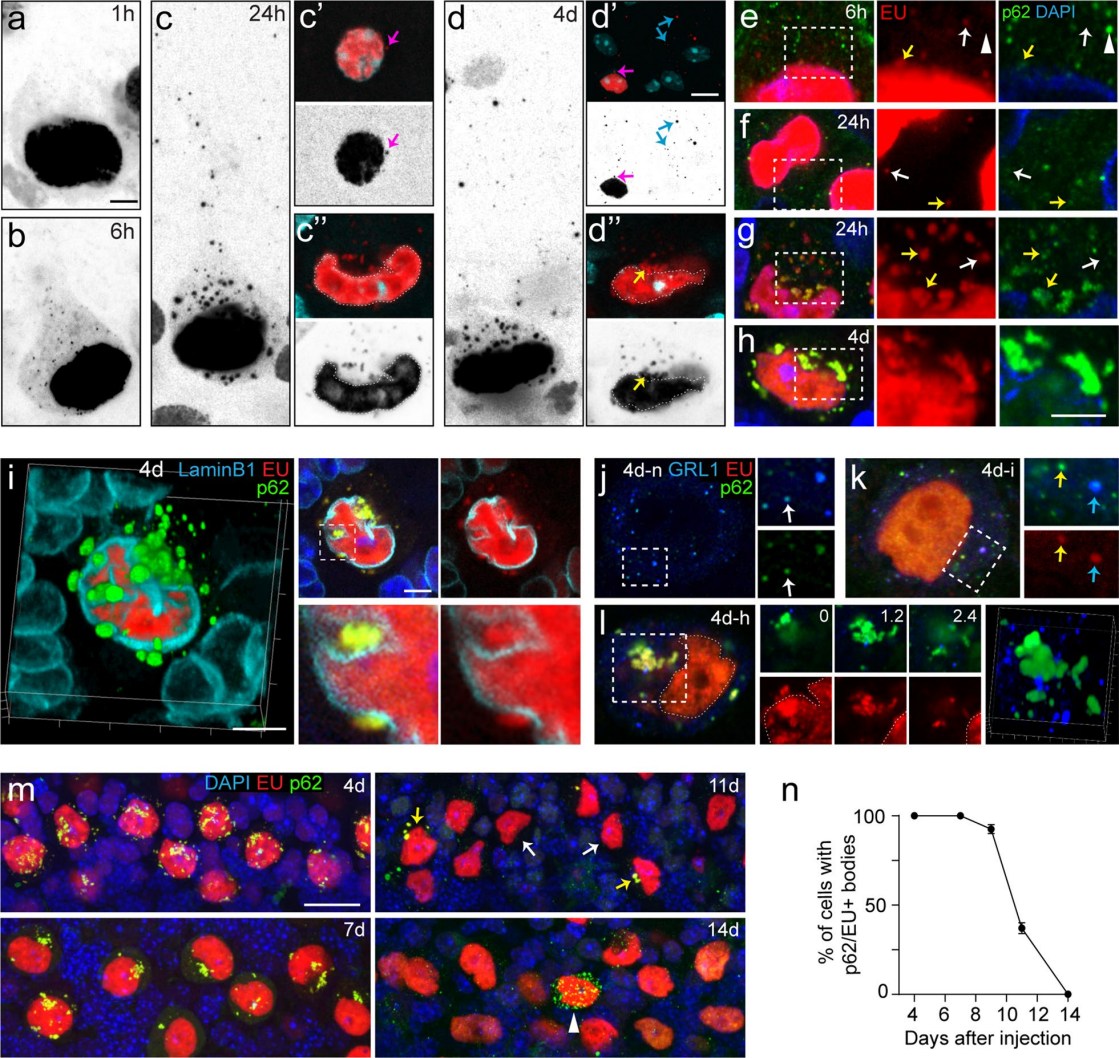
Time dependent changes of cytosolic EU labelling. **a**–**d** Inverse grey scale confocal images (maximum projection of 9 images, 4-μm stacks) illustrating EU labelling in the somato-dendritic domain of Purkinje cells (**b**–**d**) and molecular layer interneurons (pink arrows in **c′** and **d′**), and punctate labelling in the molecular layer (blue arrows in **d′**) presumably reflecting labelling in distal Purkinje cell dendrites. **c″** and **d″** are single confocal stack (1-μm optical section) of cells shown in **c** and **d**, respectively, illustrating cytoplasmic EU labelling in the perinuclear cytoplasm (yellow arrow). **e**–**h** Confocal images (1-μm optical section) illustrating co-localization of p62 immunoreactivity with cytoplasmic EU + particles (yellow arrows). Note, the prominent p62-immunoreativity in large particles in Purkinje cells after 4 days, and the difference in the size and frequency of cytoplasmic particles in 24 h Purkinje cells with intermediate (**f**) and high (**g**) EU labelling. Also note, single-labelled EU + (white arrows) and p62 + (arrow heads) particles (**e**–**g**). **i** 3D-reconstruction and confocal image of Purkinje cell after 4 days immunolabelled for p62 and LaminB1, illustrating the presence of EU + /p62 + bodies the within nuclear invaginations and in the peri-nuclear cytoplasm. **j**–**l** Triple staining for GABARAPL1 (GRL1), p62 and EU, showing the distribution of GABARAPL1 + autophagosomes in Purkinje cells after 4 days with no EU (**j**, **d**–**n**), intermediate (**k**, 4d–i) or high (l, 4d–h) EU levels. In EU-negative Purkinje cells p62 and GABARAPL1 generally co-localized (white arrows in **j**). In EU + cells GABARAPL1-immoreactivity was found in a subset of EU + /p62 + particles (cyan arrows in **k**), was absent in others (yellow arrows in **k**), and was consistently absent in large EU + /p62 + bodies (**l**). 0, 1.2 and 2.4 (in **l**) represent the z-position (in μm) of the individual images in the confocal stack. **m**, **n** Confocal images (maximum projection of 20 images, 9-μm stacks) and graph (**n**) illustrating the gradual disappearance of cytosolic EU + /p62 + bodies in Purkinje cells between 4 and 14 days. At 9 and 11 days, EU + /p62 + bodies were present in some Purkinje cells (yellow arrows), while absent in others, despite equal levels of nuclear EU labelling (white arrows); at 14 days all EU + /p62 + bodies had disappeared. Note, after 14 days, an example of a Purkinje cell filled with EU−/p62 + particles (arrow head). Values (in **n**) are derived from Purkinje cell counts in zones with high EU labelling and represent mean ± SE (n = 2 animals per time point). Scale bars: 5 μm (**a**, **h**, **i**); 10 μm (**d′**); 20 μm (**m**)

In cultured, non-neuronal cells, a substantial portion of EU redistributes from the nucleus to the cytoplasm consistent with nuclear export of EU-labelled RNA [29]. In cultured hippocampal neurons, EU labelled RNA was found to accumulate in dendrites within hours [1]. In Purkinje cells, we observed a low level of cytoplasmic EU labelling that, over time, evolved from no or minimal visible signal above background at 1 h post-injection, to a low level of diffuse and fine punctate labelling throughout the cell body and proximal dendrites at 6 h post-injection, to larger granules 24 h post-injection (Fig. 4a–c). EU + puncta were more frequent and showed higher labelling intensities in lobules with high overall EU labelling. EU + granules were present in sporadic molecular layer interneurons (Fig. 4c′), and large neurons of the cerebellar nuclei as well (data not shown). In summary, although 24 h post-injection the bulk of EU labelling is still concentrated in the nuclei, there is also cytoplasmatic labelling, primarily consisting of puncta and granules in the cell body and proximal dendrite of Purkinje cells.

We next investigated the sub-cellular distribution of in vivo EU-labelled nascent RNA beyond the time points assessed previously. In animals sacrificed 4 days post-injection, Purkinje cells still showed prominent nuclear labelling, complemented by particulate EU staining in the somato-dendritic domain (Fig. 4d). Cytoplasmic staining, in addition to punctate and granule-like particles with diameters < 0.4 μm, consisted of larger EU + bodies with various morphologies with lengths up to 4 μm (Fig. 4d). Significantly, larger EU + bodies were most abundant near the nuclear envelope and within nuclear invaginations (Fig. 4d).

Double-labelling for p62/SQSTM1 (hereafter p62), a multifunctional scaffold that operates as an autophagy receptor of various substrates [40, 58], showed that the majority of EU + particles also were labelled for p62 (Fig. 4e–h). Small cytoplasmic EU + puncta, as observed in Purkinje cells 6 h after EU injection, usually were p62-negative (Fig. 4e, f), while larger EU + granules and bodies invariably were p62-positive (Fig. 4g–i). To examine the relationship of p62 + /EU + bodies with autophagy, we double-labelled for GABARAPL1, an Atg8-homologue that is abundantly expressed in many neuronal populations, including Purkinje cells, and represents a neuronal marker for autophagosomes that in part co-localizes with p62, in particular following autophagy inhibition [37, 57]. In Purkinje cells that were not labelled for EU (in the anterior vermis distant from EU injection site), GABARAPL1-immoreactivity was localized diffusely in the cytoplasm of Purkinje cells as well as in fine granules that in part also were p62 positive (Fig. 4j), consistent with reported data [37]. A similar distribution was observed in EU-labelled Purkinje cells. GABARAPL1-immoreactivity was associated with a subset of EU + /p62 + particles (Fig. 4k). However, large EU + /p62 + bodies did not stain for GABARAPL1 (Fig. 4l). These data indicate that EU + /p62 + particles only in part represent autophagosomes.

### Transient cytosolic versus persistent nuclear EU labelling in Purkinje cells

To further examine the evolution of EU labelling in Purkinje and other cerebellar cells, we analysed EU labelling 7, 9, 11 and 14 days post-injection. Remarkably, EU labelling was still present in Purkinje cell nuclei at all these time points (Fig. 4m). EU labelling was also present in Purkinje cells in lobules with relatively low overall EU labelling. Furthermore, nuclear EU labelling persisted in Bergmann glia and a subset of molecular layer interneurons. Within Purkinje cells, cytosolic EU + /p62 + granules and larger polymorphic bodies were still present at 7, 9 and 11 days but they were absent at 14 days post-injection (Fig. 4m, n). In fact, EU + /p62 + granules were already absent in a subset of Purkinje cells at 11 days (Fig. 4m, n). These data indicate that cytoplasmic EU labelling in Purkinje cells evolves from diffuse labelling and fine granules at 6 and 24 h post-injection, to larger granules and polymorphic p62 + bodies in subsequent days, and disappears before 14 days post-injection (Fig. 4).

### High levels of EU cause Purkinje degeneration

**Fig. 5:**
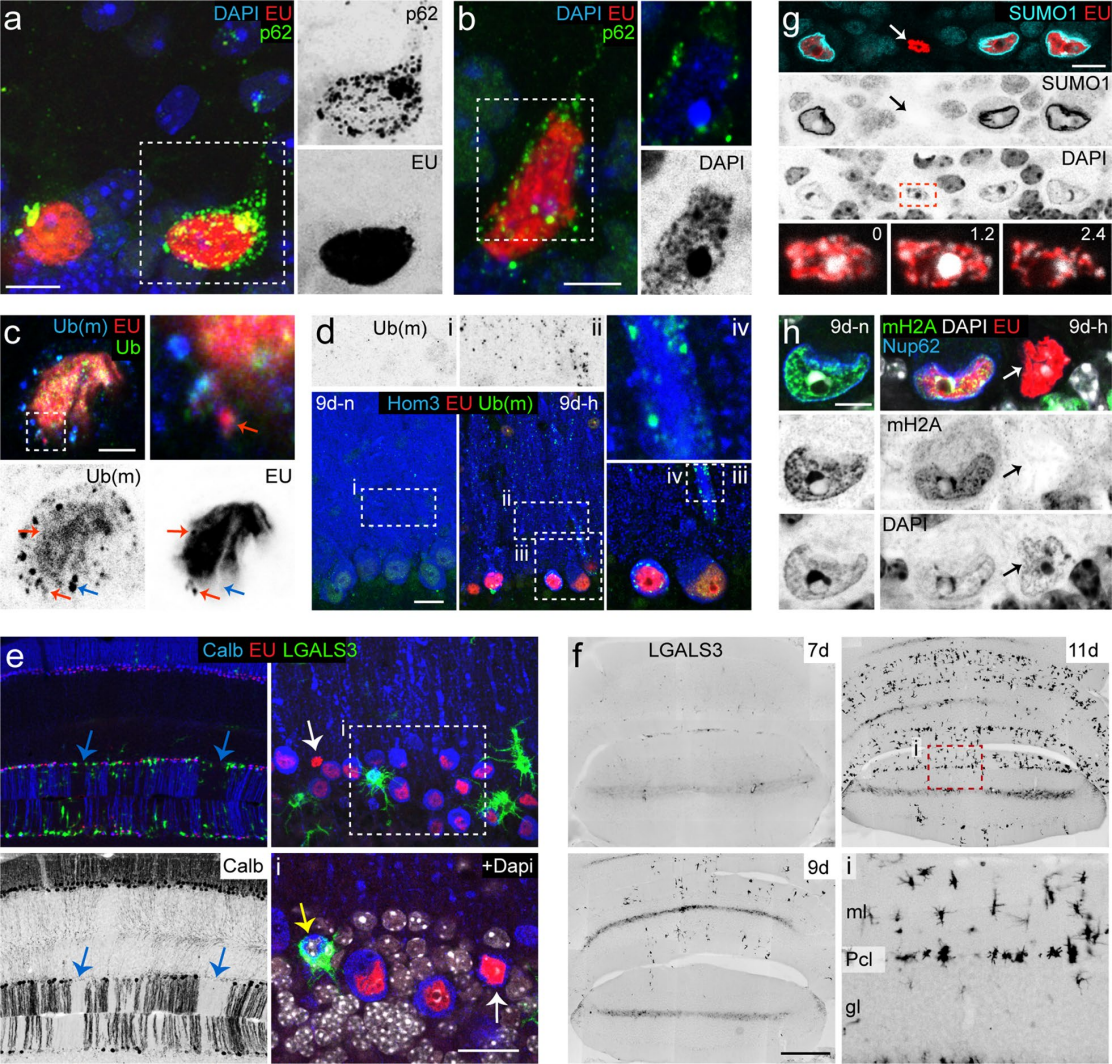
High levels of EU triggers Purkinje cell degeneration. **a**, **b** Examples of Purkinje cells with a large number of cytosolic EU−/p62 + particles. Note (in **a**) that the cell with EU−/p62 + particles also exhibits EU + /p62 + bodies, and that the Purkinje cell in **b** has an atrophied appearance and a nucleus with abnormal chromatin characterized by DAPI-dense foci. Both examples are from mice 11 days after injection. **c** Example of a Purkinje cell with ubiquitin + particles (blue arrow) stained with 2 different antibodies (Ub(m), monoclonal antibody FK2; Ub, polyclonal antibody). Cytosolic EU + particles do not stain for ubiquitin (red arrows). **d** Triple staining for ubiquitin, homer3 and EU, showed reduced homer3 staining, reflecting Purkinje cell dendritic atrophy, and ubiquitin + particles in the somato-dendritic domain of Purkinje cells in regions with high EU labelling (9d–h). Parts of the cerebellar cortex without EU labelling (9d–n) in the same mouse show normal homer3 staining and no ubiquitin + particles. **e** Low- and high-magnification confocal images illustrating the disappearance of calbindin-immunoreactivity in the molecular and Purkinje cell layers (blue arrows) indicating Purkinje cell loss 14 days after EU injection. Purkinje cell loss is associated with the appearance of LGALS3 + phagocytosing microglia cells that occasionally engulf a degenerating Purkinje cell (yellow arrow). White arrows point to atrophied Purkinje cells with shrunken strongly EU + nuclei. **f** Inverse grey scale wide-field images of caudal vermis (lobule 8 and 9) showing that while absent 7 days after EU injection, LGALS3 + microglia cells are present in the molecular and Purkinje cell layers 9 and 11 days after injection. **g**, **h** Immunolabelling for the nucleopore protein Nup62 and Sumo1 that in normal Purkinje cell strongly outline the nuclear envelope, shows that a subset of EU + Purkinje cell nuclei are devoid of nuclear envelope staining (arrows in **g**, **h**). These nuclei showed high EU levels, and a somewhat shrunken appearance and in all occasions were immuno-negative for the histone marker variant macroH2A (arrow in **h**) that in normal Purkinje cells co-distributes with heterochromatin. 0, 1.2 and 2.4 (in **g**) represent the z-position (in μm) of the individual images in the confocal stack. Scale bars: 5 μm (**b**, **c**, **h**); 10 μm (**a**, **g**); 20 μm (**d**, **e**); 200 μm (**f**)

In animals sacrificed 9, 11 or 14 days post-EU-injection, p62 immunostaining also revealed Purkinje cells with a large number of EU−/p62 + particles that were distributed throughout the soma and the proximal dendrite (Figs. 4m, 5a). We observed these cells only in lobules with high EU labelling, and usually they contained some EU + /p62 + particles (Fig. 5a). Occasionally, cells with EU−/p62 + particles showed an atrophied, degenerative appearance (Fig. 5b). Immunostaining for poly-ubiquitinated epitopes showed that EU−/p62 + particles were strongly immunoreactive for ubiquitin, in contrast to EU + /p62 + structures that were not or weakly immunoreactive for ubiquitin (Fig. 5c,d). In animals sacrificed 9, 11 or 14 days post-EU-injection, ubiquitin-immunoreactive particles were also distributed throughout the molecular layer where they co-localized with Purkinje cell markers such as calbindin and homer3 (Fig. 5d). Staining for the Purkinje cell markers calbindin and homer3 also revealed Purkinje cell degeneration and death resulting in either reduced or no staining of these markers in parts of the molecular layer (Fig. 5d, e). The occurrence of Purkinje cell degeneration is illustrated by immunostaining for LGALS3 (galectin3 formerly known as Mac2) that in the central nervous system outlines activated phagocytosing microglia as well [12]. LGALS3 + microglia were not observed in the cerebellum 4 or 7 days after EU injection, occurred at low numbers after 9 days, and were more frequent after 11 and 14 days (Fig. 5e, f). LGALS3 + microglia cells were concentrated in the molecular and Purkinje cell layers (Fig. 5e, f), and their presence correlated with loss of calbindin staining (Fig. 5e). Occasionally, we noted severely atrophied calbindin + cells that were enwrapped by LGALS3 + microglia (Fig. 5e), presumably representing Purkinje cells in a final stage before disappearance.

To examine whether degeneration of EU-labelled Purkinje cells is associated with activation of apoptotic pathways, we stained for active caspase3. Although we found a few active caspase3 + cells with pyknotic nuclei indicative of apoptosis in the molecular and granule cell layers (Additional file 1: Figure S1), we never found caspase3 + Purkinje cells. Also, Purkinje cells with an atrophied EU + nucleus as shown in Fig. 5b in all instances stained negative for active caspase3. Instead, we found that Purkinje cells with atrophic nuclei show severely reduced or no staining for nucleopore markers like Nup62, SUMO1 (Fig. 5g, h) and RANGAP1, as well as for the nuclear lamina protein laminB1 (data not shown), indicating that they are in a defective state. However, DAPI staining showed that the chromatin organization in Purkinje cells with atrophied nuclei appears relatively normal, although they show an increased number of DAPI-dense foci (Fig. 5b, h). Furthermore, they generally were not surrounded by activated LGALS3 + microglia (Fig. 5e). To further characterize the atrophied nuclei, we immunostained for macroH2A, a histone variant that is primarily associated with heterochromatin [67] and in nuclei of aged Purkinje cells may develop a multifocal granular distribution [30]. Remarkably, Purkinje cells with atrophied EU + nuclei in all instances were completely devoid of macroH2A-immunoreactivity (Fig. 5h). Together, these data indicate that EU may trigger Purkinje cell death, characterized by a distinctive morphological and molecular profile. It is beyond the scope of the present study to fully demarcate the identity of Purkinje cell degeneration triggered by EU, but the high level of p62 + /Ubi + particles point to an important role of autophagy.

### Reduced EU labelling in Purkinje cells of DNA-repair deficient mouse model

Having established that we can obtain reproducible EU labelling in cerebellar cells, we evaluated our approach in two Purkinje cell-specific mutant mouse models that display adult-onset of Purkinje cell degeneration, and may exhibit decreased RNA production. We first examined *Ercc1*^*d/f*^*Pcp2-Cre* mice with Purkinje cell-specific deficiency of ERCC1, a protein that in a complex with XPF is a critical endonuclease in multiple DNA repair pathways, including transcription-coupled and global genome nucleotide excision repair [36, 71, 76]. Global ERCC1-deficient mice exhibit a short life span and progressive degenerative changes in multiple tissues including the central nervous system [12, 27, 72], and show a gene length dependent reduction in transcription [70] and reduced EU staining in liver cells [39]. We examined *Ercc1*^*d/f*^*Pcp2-Cre* mice for EU labelling at 6 months of age, when they show cerebellar motor deficits and Purkinje cell loss [11].

**Fig. 6:**
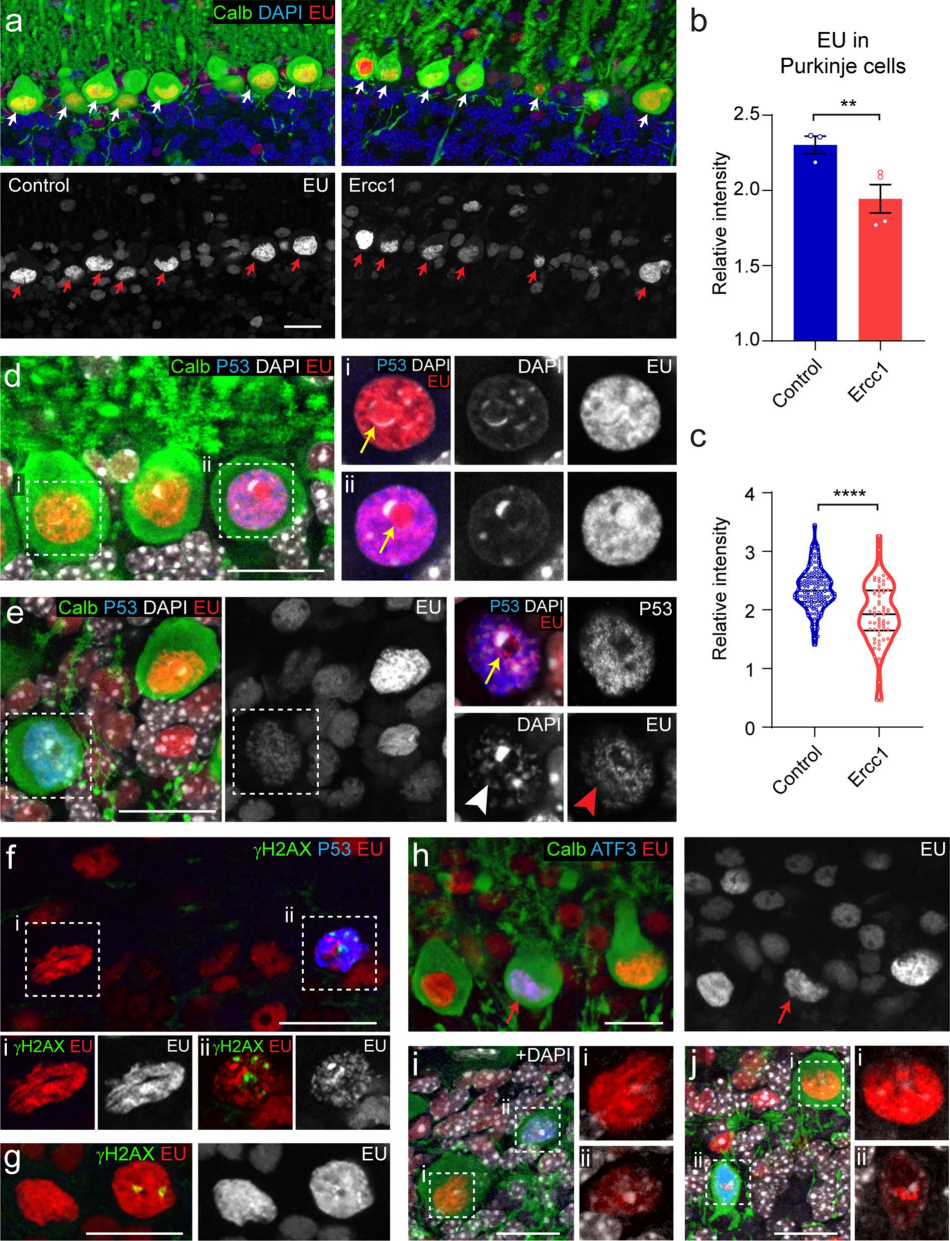
Reduced EU-labelling in Purkinje cells of a DNA-repair deficient mouse model. **a**–**c** Confocal images (maximum projection of 10 optical sections, 4.5-μm stacks) and graphs demonstrating reduced and more variable EU labelling in Purkinje cells from 6-months-old *Ercc1*^*d/f*^*Pcp2-Cre* (Ercc1) mice. Animals were perfused 1 h following EU injection. Purkinje cells (arrows) were identified with calbindin immunostaining. Bar graph in **b** shows the means ± SE of relative EU labelling intensities in Purkinje cells per animal (n = 3 and 4 for control and Ercc1, respectively, with at least 10 cells per mouse). Values from individual Purkinje cells are calibrated for the mean of labelling intensities of surrounding Bergman glia cells, and are shown in the violin plot in **c**, with the black lines indicating the median and the interquartile range (25–75% data range). The contour line represents the 95% confidence interval. Note the overall reduced nascent RNA labelling by EU and increased variation in Ercc1 Purkinje cells (**c**). **b *P* < 0.01 (unpaired Student’s t-test); ****c *P* < 0.0001 (Welch t-test). **d**–**e** Triple staining of EU, p53 and calbindin in *Ercc1*^*d/f*^*Pcp2-Cre* cerebellum, illustrating a p53 + Purkinje cell with normal EU labelling (**d**), and a p53 + Purkinje cell with strongly reduced EU labelling in both the nucleolus (arrow in **e**, compare with cells in **d**) and the rest of the nucleoplasm (arrow head in **e**). **f**, **g** Triple staining of EU, p53 and yH2AX, illustrating a p53 + Purkinje cells with yH2AX foci (**f**) with low EU labelling, and a p53- Purkinje cells with yH2AX foci (**g**) and normal EU labelling. **h**–**j** Examples of ATF3 + Purkinje cells with relatively normal EU (**h**) and reduced (**i**, **j**) EU labelling. Scale bars: 20 μm

EU staining in Purkinje cells of *Ercc1*^*d/f*^*Pcp2-Cre* mice was more variable than in control Purkinje cells, ranging from the same intensities as in control Purkinje cells to intensities equal or lower than surrounding Bergmann glia that showed moderate EU staining as in controls (Fig. 6a). Reduced labelling in a subset of Purkinje cells resulted in a lower mean relative labelling in *Ercc1*^*d/f*^*Pcp2-Cre* Purkinje cells (Fig. 6b, c). To examine whether ERCC1-deficient Purkinje cells with lower EU labelling also display signs of genotoxic stress, we double-labelled for DNA damage signalling factors p53, ATF3 and γH2AX that are all linked to repression of transcription [15, 36], and are expressed in a subset of neurons in ERCC1 mice and other DNA-repair deficient mice [4, 12, 26, 27, 34, 36, 70]. All three markers were identified in a small subset of Purkinje cells (< 1–3%), and did not consistently correlate with changes in EU labelling, although the majority of double-labelled cells did show reduced EU staining (Fig. 6d–j). In cases of p53 + Purkinje cells, we found that about half (19 of 40 cells) showed normal EU levels (Fig. 6d). P53 + cells, with reduced EU staining in general, showed abnormal EU distribution throughout the nucleus, including the nucleolus, and frequently showed abnormal DAPI staining Fig. 6e). Purkinje cells with γH2AX + foci, indicative of double-strand DNA breaks, were infrequent in our material and mostly (8 of 10) showed strongly reduced EU signals, and also stained positive for p53 (Fig. 6f). However, we also found occasional Purkinje cells with γH2AX + foci with normal EU staining (Fig. 6g). In cases of ATF3 + Purkinje cells, we found that about one-third (12 of 40 cells) showed normal EU levels (Fig. 6h). ATF3 + Purkinje cells with reduced EU staining in many occasions were smaller and showed abnormal granular DAPI staining (Fig. 6i, j). Together, the data indicate that *Ercc1*^*d/f*^*Pcp2-Cre* Purkinje cells in part show reduced EU levels and that changes in EU labelling in part coincide with the expression of genotoxic stress factors.

### Reduced EU labelling in Purkinje cells of a mouse model of SCA1

**Fig. 7:**
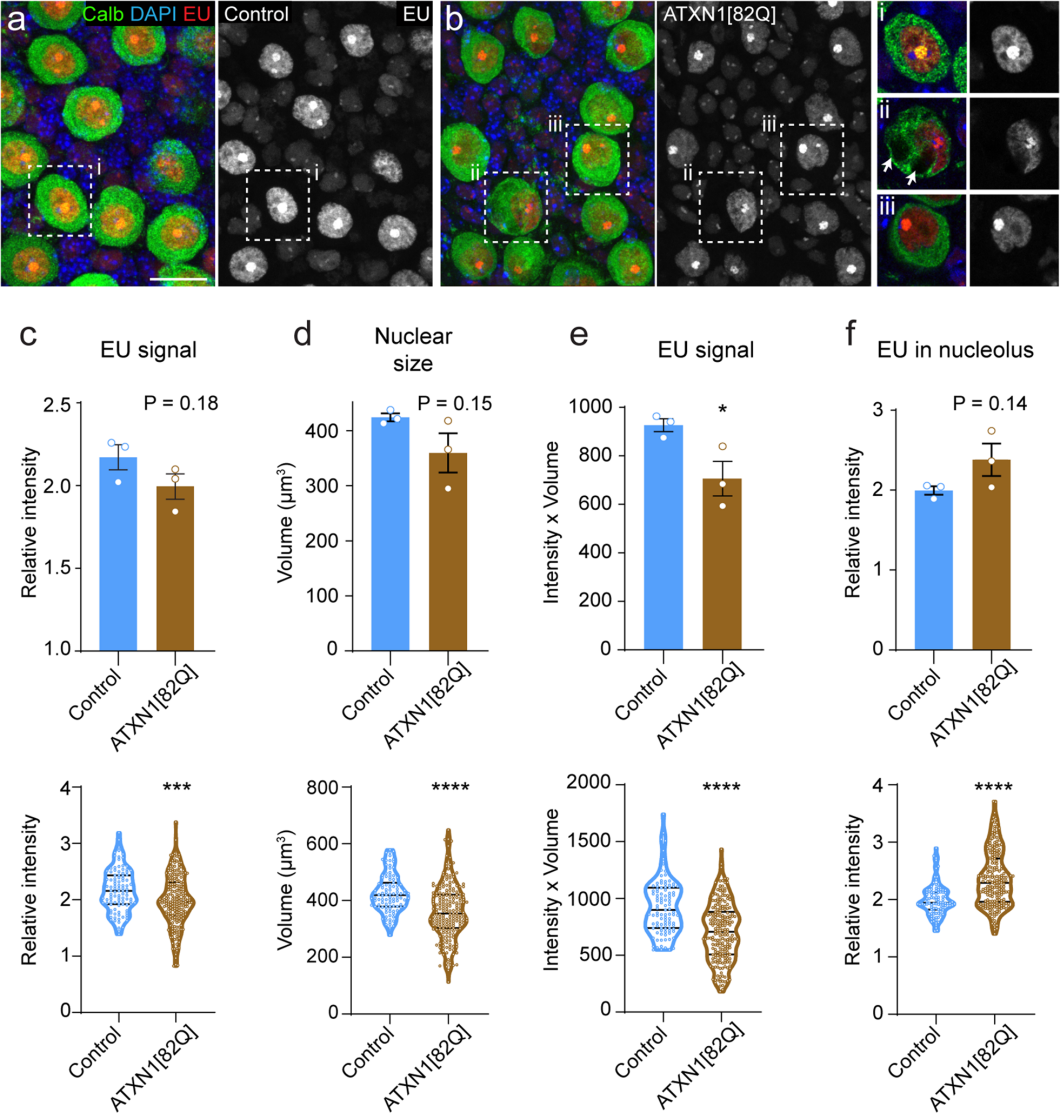
Reduced EU-labelling in Purkinje cells of ATXN1[82Q] mice. **a**, **b** Confocal images (maximum projection of 10 optical sections, 4.5-μm stacks; inserts are from single optical sections) illustrating EU labelling in Purkinje cells and Bergmann glia from 9-week-old control and ATXN1[82Q] mice. Animals were perfused 1 h following EU injection. Note that the orientation of the images is parallel to the Purkinje cell layer. Also note large vacuoles in a ATXN1[82Q] Purkinje cell (arrows in insert ii). **c**–**f** Graphs showing relative intensities of nuclear EU labelling (**c**), nuclear volume (**d**), relative EU intensity × nuclear volume (**e**); and intensity of nucleolar EU labelling relative to EU intensity of the rest of the nucleoplasm (**f**) in control and ATXN1[82Q] Purkinje cells. Bar graphs (upper row) show means ± SE per animal (n = 3). P values are from 2-tailed Student's t-tests. *P < 0.05. Violin plots show values of individual Purkinje cells. Inside the violin plots, the black lines indicate the median of the data and the interquartile range (25–75% data range). The contour line represents the 95% confidence interval. ***P < 0.001; ****P < 0.0001; Welch t-tests. Scale bars: 20 μm

We next examined EU labelling in a second mouse model that features Purkinje cell degeneration. ATXN1[82Q] transgenic mice express human ATXN1 with a polyglutamine repeat expansion specifically in Purkinje cells [10]. ATXN1[82Q] mice were examined for EU labelling at the age of 9 weeks. At this age, they show somato-dendritic atrophy of Purkinje cells [10] in association with dysregulated gene expression [24, 56]. EU labelling in Purkinje cells of ATXN1[82Q] mice showed a normal appearance (Fig. 7a, b). The relative labelling intensity in Purkinje cells compared to surrounding Bergmann glia, however, was reduced compared to controls, reaching statistical significance when comparing pooled data from individual Purkinje cells from different mice (Fig. 7c), and when correcting for the smaller nuclear size of ATXN1[82Q] Purkinje cells (Fig. 7d, e). Additionally, the ratio of labelling intensity in the nucleolus versus the rest of the nucleoplasm was altered in ATXN1[82Q] mice (Fig. 7f). Thus, EU labelling suggests reduced overall transcription in ATXN1[82Q] Purkinje cells.

## Discussion

Fluorescent staining of newly transcribed RNA via metabolic labelling with 5-ethynyluridine (EU) and subsequent click chemistry, has been used in multiple studies to visualize global transcription in cells [1, 8, 23, 29, 39, 41, 44, 52, 64, 79]. Here, we show that injection of EU directly in the cerebellum results in reproducible labelling of cerebellar neurons and glia, and in consistent cell type-specific differences in relative labelling intensities, such as Purkinje cells displaying the highest levels of labelling. We also found reduced EU labelling intensities in Purkinje cells in two mouse models of Purkinje cell degeneration. These data indicate that EU labelling can be used to probe differences and changes in global transcription in the nervous system in vivo.

Our data are consistent with in vivo [^3^H]uridine labelling studies showing rapid accumulation of radiolabeled RNA in neuronal nuclei following intracranial, intravenous or subcutaneous injection, reaching a plateau within 30–40 min after injection [16, 66, 75]. In large neurons, nuclear [^3^H]uridine labelling persisted for 24 h and showed a gradual reduction in subsequent days, while in other cell types (choroid plexus, liver) labelling disappeared more quickly [16, 75]. In our study, we found different temporal EU staining profiles within the same neuronal tissue depending on cell type, and varying from disappearance of detectable staining within 6 h post-injection in cerebellar granule cells to staining that persisted for longer than 2 weeks in Purkinje cells, Bergman glia and molecular layer interneurons. Rapid disappearance of nuclear EU staining in granule cells is consistent with the rapid drop of EU staining in pulse-chase experiments with cultured 3T3 cells [29]. These pulse-chase experiments led to the conclusion that EU pulses shorter than 3 h are too short to significantly label stable RNA species, whereas 24 h EU labelling results in nuclear and cytoplasmic staining that persists for more than 24 h because of labelling of stable RNA species [29].

Our finding that a significant amount of nuclear EU staining persisted in Purkinje cells for more than 2 weeks, contrasts with [^3^H]uridine labelling studies showing minimal neuronal labelling after 12 days [49], as well as with experiments in cultured cells showing that after EU labelling for 24 h, nuclear staining dropped to low levels after 3 h, while higher levels of labelling persisted in the cytoplasm for more than 24 h [29]. A minor portion of newly transcribed RNA is known to be transported to the cytoplasm (about 50% of RNA polymerase 1 products and 5% of RNA polymerase 2 products), while a major portion is rapidly degraded in the nucleus (e.g. intronic RNAs), and a yet incompletely defined populations of RNA may remain in the nucleus for longer times [14, 28, 32, 47, 69, 78]. Persistent nuclear EU labelling also has been observed in the snail nervous system, which showed a population of strongly labelled glia cells and neurons more than 2 months after bathing juvenile animals for 4 h in 1 mM EU [22]. This persistent labelling was proposed to reflect long living RNA species specific for snail nervous system [22]. Another possibility is that prolonged labelling result from cycles of renewed integration of EUMP ribonucleotides, derived from RNA degradation, into nascent RNAs. Alternatively, prolonged nuclear labelling results from non-physiological properties that, for instance, impair the degradation of EU-labelled RNA species [29, 69].

Studies with modified uridines, such as bromo-uridine (BrU), fluoro-uridine (FU) and 4-thiouridine (4sU) provided evidence for cytotoxicity and altered properties of labelled RNA species [6, 21, 61, 63, 69, 74, 81]. Toxic mechanisms include defective splicing, impaired pseudo-uridylation, and altered stability of modified RNA's [6, 21, 61, 63, 74, 81]. Similar mechanisms can be envisaged for EU labelled RNA species, depending on dosage and degree of incorporation of EU in nascent RNA. In cells incubated 24 h with 1 mM, EU was estimated to label about 6% of uridine residues in polyA RNA [29], and showed no major effect on mRNA transcription and processing in studies using similar conditions [14, 62]. Accordingly, no toxic effects were reported in HeLa cells following an 18-h incubation with 1 mM EU [20]. However, at least two studies have documented toxic effects of EU: prolonged incubation with EU (24–48 h) reduced viability of A549 [69] and HEK293 cells [42] at concentrations of 400 μM (for 48 h) and 1 mM EU (for 24 h), respectively. In accordance with toxic properties of EU labelled RNA, we found that high levels of EU cause slow Purkinje cell degeneration resulting in their death and disappearance from around 9 days post-injection. Purkinje cell degeneration showed unique features such as the disappearance of nuclear envelope and chromatin markers, and was associated with high level of p62 + Ubi + particles, indicative of a role of autophagy.

In addition to Purkinje cell degeneration, we observed a second major abnormality in strongly EU-labelled Purkinje cells: they developed EU + cytoplasmic structures that co-stained for the autophagy adaptor p62, and that evolved from fine granules at 6 and 24 h post-injection to larger structures in subsequent days (Fig. 4). The accumulation of EU labelling in p62 + bodies indicates that cytoplasmic RNA species are directed to the autophagy-lysosome degradation pathways [40] suggesting that they cannot be normally degraded. We found that only part of EU + /p62 + particles stained for GABARAPL1, an Atg8-homologue that represents a marker for autophagosomes in Purkinje cells and other populations of neurons [37, 57]. Both the absence GABARAPL1 immunoreactivity in a large portion of EU + /p62 + particles, and their accumulation in time, may suggest that the amount of EU + /p62 + material exceeds the capacity of the autophagic pathway.

EU + /p62 + particles also occurred in dendrites of Purkinje cells (Fig. 4), consistent with the notion that a portion of messenger RNA and ribosomes is in the dendritic compartment of Purkinje cells [80]. Interestingly, particulate dendritic EU labelling was also found in primary cultured rat hippocampal neurons [1]. In this study, dendritic EU + particles co-stained for mRNA binding proteins and ribosomal RNA markers [1] and it remains to be determined whether they represent the same non-physiological p62 structures observed in our study in Purkinje cells. A question that remains to be addressed is how the appearance of cytoplasmic EU + /p62 + bodies is connected to Purkinje cell degeneration. EU + /p62 + bodies are most dominant 4–7 days post-injection, while Purkinje cell atrophy and death occurs later and seemingly in a smaller subset of Purkinje cells. It is also important to note that prolonged nuclear EU labelling also occurs in Purkinje cells that express relatively low levels of EU and populate parts of the cerebellar cortex that never showed cytoplasmic p62 structures.

A main goal of our study was to determine whether we could measure changes in EU labelling in mouse models of neurodegenerative diseases. EU labelling had been shown to be particularly useful to measure reduced RNA synthesis after genotoxic insults, such as UV-irradiation, and to assess defective recovery of RNA synthesis in cellular models of DNA repair deficiency [4, 8, 39, 43, 44, 79]. We, therefore, first examined a Purkinje cell-specific ERCC1-deficient mouse model that displays a defect in multiple DNA repair pathways including global genome and transcription-coupled nucleotide excision repair selectively in Purkinje cell, and shows gradual adult-onset Purkinje cell degeneration and death [11, 76]. We only analysed animals at a single age (6 months) when Purkinje cells already had started to die, and we found more variable EU labelling than in control Purkinje cells. Labelling ranged from the same labelling as in control Purkinje cells to considerably lower levels in a subset of Purkinje cells. The conclusion that can be drawn from these data is that even following the onset of Purkinje cell degeneration, only a subset of ERCC1-deficient Purkinje cells experiences global down-regulation of transcription. Significantly, Purkinje cells with strong nuclear labelling of stress DNA damage signalling factors p53 and ATF3 in part displayed normal EU labelling. Our findings in Purkinje cells differ from those in ERCC1-deficient liver, where a robust decline in transcription occurred in the bulk of liver cells [39]. In complex with XPF, ERCC1 is a critical endonuclease in various DNA repair processes, in particular transcription-coupled repair, and accordingly ERCC1-deficiency affects transcription via the accumulation of transcription blocking lesions that may impair transcription of vital genes, or cause the production of abnormal transcripts [36, 39, 43, 70, 76]. In line with the stochastic nature of endogenous transcription blocking DNA lesions, ERCC1-deficiency has been found to more severely affect long genes (> 50 kbase) as compared to short genes, a phenomena observed in accelerated aged ERCC1 mouse liver and normally aged rat liver and human brain [39, 70]. The stochastic nature of endogenous DNA lesions also predicts that different vital genes are affected in different cells, resulting in different modes of degeneration and cell death. Such a variety of cell death mechanisms is consistent with our neuropathological findings in ERCC1- and other transcription-coupled repair-deficient mice [4, 5, 12, 27, 70], and with the heterogeneity of EU labelling in ERCC1-deficient Purkinje cells observed in this study. Also in ERCC1-deficient liver there is down-regulation of transcription and increased heterogeneity between individual hepatocytes. DNA-damage-induced transcriptional stalling may also trigger a genome-wide down-regulation of transcription, where transcription stalling in a limited number of genes triggers a genome-wide down-regulation of transcription [36, 39, 46]. Such a DNA damage response globally affecting transcription also may operate in a subset of ERCC1-deficient Purkinje cells, for instance the Purkinje cells expressing ATF3, a transcription factor that can be induced by DNA damage to reduce the expression of large sets of genes [15, 36]. Significantly, ATF3 + Purkinje cells with reduced EU staining in many occasions were smaller suggesting that global transcription repression in Purkinje cells is associated with somato-dendritic atrophy. Likewise, neuronal atrophy in association with DNA damage and transcription inhibition was found in cultured dorsal root ganglion neurons treated with oxaliplatin, a genotoxin that generates transcription- (and replication-) blocking lesions [79]. Transcriptional repression in conjunction with somatodendritic atrophy also is suggested by reduced EU-labelling in the ATXN1[82Q] mice. In this mouse model, the aberrant interaction of expanded polyQ-ATXN1 with the transcriptional repressor capicua (CIC) is shown to cause down-regulation of a large number of Purkinje cell genes [24, 56], which as suggested by our EU data may result in reduced overall transcription.

In conclusion, in the present study we show that reproducible labelling of nascent RNA in neurons in vivo can be achieved via direct intraparenchymal EU injections in brain tissue. Our approach provides an alternative for systemic intraperitoneal injections of EU [29] or fluoro-uridine [2, 9], and results in superior in vivo labelling of nascent RNA in nervous system cells. As shown in this study, EU labelling can be easily combined with immunofluorescence to correlate labelling of nascent RNA with neuropathological markers. This combination may be particularly valuable for examining changes in transcription in mouse models for nervous system disorders linked to abnormalities in RNA metabolism [48, 78]. Finally, our EU labelling approach can also be used for isolation and sequencing of nascent RNA from neuronal populations in vivo [77] and as such provides a valuable research tool for better studying nascent RNA dynamics in vivo in neurons.

## Supplementary Information

**Additional file 1:** Fig. S1. No caspase3+ cells in the Purkinje cell layer. Triple staining for active caspase 3, calbindin and EU, showing a caspase3+ cell with a pyknotic nucleus (arrow) in the molecular layer 14 days after EU injection.

## Data Availability

The datasets obtained and analysed during the current study are available from the corresponding author on reasonable request.
