# Supplementary material for: In vivo 5-ethynyluridine (EU) labelling detects reduced transcription in Purkinje cell degeneration mouse mutants, but can itself induce neurodegeneration
Source: Acta Neuropathol Commun. 2021 May 21;9:94. doi: 10.1186/s40478-021-01200-y (PMC8139001; doi:10.1186/s40478-021-01200-y)
Supplement: Supplementary file 1 — Additional file 1: Fig. S1. No caspase3+ cells in the Purkinje cell layer. Triple staining for active caspase 3, calbindin and EU, showing a caspase3+ cell with a pyknotic nucleus (arrow) in the molecular layer 14 days after EU injection. [file 40478_2021_1200_MOESM1_ESM.docx]

Online Resource


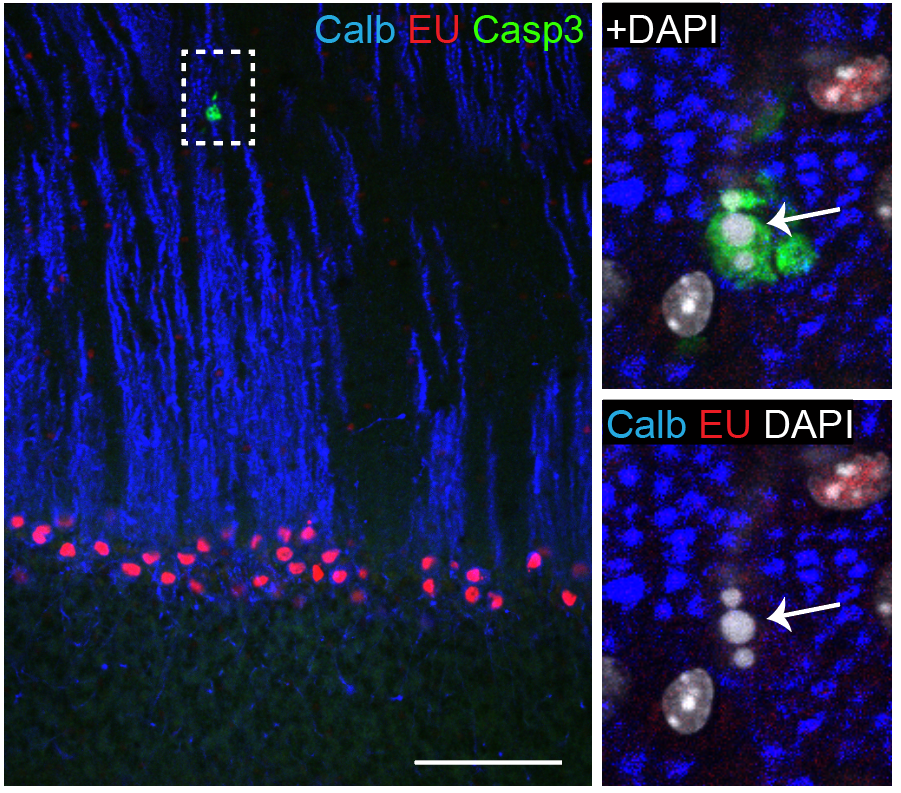


Figure 1

Triple staining for active caspase 3, calbindin and EU, showing a caspase3+ cell with a pyknotic nucleus (arrow) in the molecular layer 14 days after EU injection. Caspase3+ cells never occurred in the Purkinje cell layer.

Scale bar: 100 μm
